# Translation, Cultural Adaptation, and Psychometric Validation of the Iranian Version of the Eating Behavior Assessment Questionnaire for Obesity (EBA-O) in Adults with Obesity and Overweight

**DOI:** 10.3390/nu18030454

**Published:** 2026-01-30

**Authors:** Maryam Mohamadinarab, Atoosa Saidpour, Pegah Rahbarinejad, Parisa Amiri, Mir Saeed Yekaninejad, Fereshteh Sadat Hosseinian Ghamsari, Marianna Rania, Cristina Segura-Garcia, Abdolreza Norouzy, Mohammad Safarian

**Affiliations:** 1 Department of Nutrition, Faculty of Medicine, Mashhad University of Medical Sciences, Mashhad 9177948564, Iran; mohammadinm992@mums.ac.ir (M.M.); rahbarinp991@mums.ac.ir (P.R.); 2 National Nutrition and Food Technology Research Institute, Shahid Beheshti University of Medical Sciences, Tehran 1985717413, Iran; atoosa.saidpour@gmail.com; 3 Research Center for Social Determinants of Endocrine Health & Obesity Research Center, Research Institute for Endocrine Sciences, Shahid Beheshti University of Medical Sciences, Tehran 1985717413, Iran; parisaamiri@yahoo.com; 4 Department of Epidemiology and Biostatistics, School of Public Health, Tehran University of Medical Sciences, Tehran 1985717413, Iran; yekaninejad@yahoo.com (M.S.Y.); f.hoseinian6442@gmail.com (F.S.H.G.); 5 Psychiatry Unit, Outpatient Unit for Clinical Research and Treatment of Eating Disorders, University Hospital Renato Dulbecco, 88100 Catanzaro, Italy; marianna.rania@hotmail.it; 6 Department of Medical and Surgical Sciences, University Magna Graecia of Catanzaro, 88100 Catanzaro, Italy; segura@unicz.it; 7 Department of Clinical Nutrition, Faculty of Medicine, Iran University of Medical Sciences, Tehran 1416634793, Iran

**Keywords:** obesity, eating behavior, assessment, persian validation, psychometric properties

## Abstract

**Background:** Research has shown that disordered eating behaviors—including binge eating, night eating syndrome, and food addiction—contribute to the heterogeneity of obesity and assist in phenotyping patients for more tailored interventions. The Eating Behavior Assessment for Obesity (EBA-O) is a recently developed 18-item questionnaire that assesses five pathological eating-behavior domains among individuals with obesity (night eating, food addiction, sweet eating, hyperphagia, and binge eating). The present study aimed to translate, culturally adapt, and validate the Persian (Farsi) version of the EBA-O. **Methods:** The original English EBA-O was translated into Persian following a standardized forward–backward translation procedure, with cultural adaptations implemented to ensure linguistic accuracy and conceptual clarity. A cross-sectional sample of 278 Iranian adults with overweight or obesity (body mass index [BMI] ≥ 25 kg/m^2^) completed the Persian EBA-O. Confirmatory factor analysis (CFA) was conducted to verify the five-factor model in the Persian sample. Internal consistency was evaluated using Cronbach’s alpha and composite reliability (CR). Convergent validity was assessed using the average variance extracted (AVE), and discriminant validity was examined with the Heterotrait–Monotrait ratio (HTMT). Model fit indices, including the Comparative Fit Index [CFI], Tucker–Lewis Index [TLI], Normed Fit Index [NFI], Goodness-of-Fit Index [GFI], the Standardized Root Mean Square Residual [SRMR] and relative chi-square value [χ^2^/*df*] were used to determine the adequacy of the factor structure. **Results:** The Persian EBA-O demonstrated a clear and stable five-factor structure consistent with the original instrument. CFA indicated good model fit (CFI = 0.95, TLI = 0.94, NFI = 0.91, GFI = 0.92, SRMR = 0.05, χ^2^/*df* = 1.94), confirming the presence of the intended domains. Internal consistency was acceptable to high across all subscales (Cronbach’s α = 0.78–0.86; CR > 0.70), and the total scale showed strong reliability. Three of the five factors demonstrated acceptable convergent validity (AVE = 0.54–0.68), while Food Addiction (AVE = 0.46) and Night Eating (AVE = 0.43) fell slightly below the 0.50 threshold; however, their adequate CR and α values indicate that these constructs remain coherent and psychometrically sound. All inter-factor correlations satisfied discriminant validity criteria (HTMT < 0.90), with the highest association observed between the Binge Eating and Hyperphagia factors. Overall, the psychometric properties of the Persian EBA-O were comparable to those reported in the original validation and subsequent translations. **Conclusions:** The Persian version of the EBA-O is a valid and reliable instrument for assessing pathological eating behaviors among individuals with obesity. It preserves the original questionnaire’s five-factor structure and demonstrates acceptable internal consistency and construct validity in a Persian-speaking population. This validated tool will support both clinical assessment and research on eating-behavior phenotypes and may contribute to the development of more personalized and effective obesity-management strategies among Persian-speaking individuals.

## 1. Introduction

Obesity, defined as a body mass index (BMI) ≥ 30 kg/m^2^, has shown a marked rise in prevalence over the past three decades [1]. This global increase has been strongly linked to heightened risks of a wide range of chronic diseases, including diabetes, cardiovascular disease, dyslipidemia, inflammatory disorders, depression, and cancer [2,3,4]. In Iran, similar to many countries, the prevalence of excess weight has risen substantially, with recent estimates indicating that more than one-fifth of Iranian adults are living with obesity [5].

High treatment-dropout rates in obesity care have been partly attributed to the challenge of addressing the diverse eating-behavior patterns that contribute to weight gain. A growing body of evidence suggests that identifying specific disordered eating behaviors in individuals with obesity may improve patient phenotyping and support more tailored intervention strategies [6]. Certain pathological eating patterns, such as binge eating, night eating syndrome (NES), and addictive-like eating (food addiction), are particularly prevalent among subsets of individuals with obesity and carry significant clinical implications [7].

For example, binge-eating disorder (BED) affects a notable subgroup of patients with obesity; individuals with BED have a significantly greater likelihood of being obese compared with those without the disorder [8]. Night eating syndrome is observed in approximately 6–16% of people with obesity and is associated with disrupted circadian eating patterns and insomnia, factors that can hinder weight management [9]. Likewise, food addiction, characterized by compulsive consumption of high-fat/high-sugar foods, has been reported in about one in six individuals with overweight or obesity, suggesting that a sizeable subgroup may experience an addictive drive toward food intake [10].

These eating behaviors frequently overlap and contribute to the phenotypic heterogeneity of obesity, underscoring the importance of systematically assessing them as part of comprehensive obesity profiling. Phenotyping individuals according to their disordered eating behaviors may enable clinicians to implement more targeted behavioral or pharmacological interventions.

Validated questionnaires are currently available to measure several of these pathological behaviors individually—for example, the Binge Eating Scale for binge eating, the Night Eating Questionnaire for NES, and the Yale Food Addiction Scale for food addiction. However, administering multiple separate instruments can be time-consuming and impractical in routine clinical settings [11]. To address the need for a comprehensive yet efficient tool, Segura-García et al. [12] developed the Eating Behavior Assessment for Obesity (EBA-O). This 18-item self-report instrument simultaneously evaluates five pathological eating-behavior domains common in obesity: night eating, food addiction, sweet eating (excessive consumption of sweets in response to emotional cues), hyperphagia (habitual consumption of unusually large meal portions), and binge eating [12].

Each item is rated on an 8-point frequency scale (0 = “never” to 7 = “every day”), referring to the past three months. The EBA-O yields five subscale scores plus a total score, with higher values indicating more frequent pathological eating patterns. A score of 4 or higher on any subscale or the total score is considered clinically significant. The main advantage of the EBA-O is its ability to assess multiple facets of eating pathology within a single brief instrument, eliminating the need for multiple questionnaires.

Additionally, the EBA-O was designed to be easy to administer and score, allowing use by healthcare providers without specialized expertise in eating disorders [11]. In its initial validation, the EBA-O demonstrated strong psychometric properties: exploratory factor analysis identified the expected five factors, explaining approximately 68% of response variance, and all subscales showed high internal consistency (McDonald’s ω = 0.80–0.92) [12]. Confirmatory factor analysis in a clinical obesity sample further supported the five-factor model, yielding acceptable fit indices (e.g., CFI = 0.93, TLI = 0.92, RMSEA = 0.075) and significant correlations with related psychopathology measures, establishing convergent validity [12].

A Greek-language validation by Mavrandrea et al. [11] confirmed that the second-order five-factor structure remained robust in a Greek clinical population, demonstrating excellent model fit (CFI = 0.99, RMSEA = 0.03) and high reliability (total scale ω = 0.94). The Greek subscales were strongly intercorrelated and showed solid concurrent validity with measures such as the Eating Disorder Examination Questionnaire and the Yale Food Addiction Scale. These findings highlight the cross-cultural robustness and clinical utility of the EBA-O.

Despite these advancements, no validated Persian (Farsi) version of the EBA-O has been available to date. Persian is spoken by over 80 million people, making the availability of psychometrically sound assessment tools in this language essential for both research and clinical practice. Cultural and linguistic factors can influence how eating-related psychopathology is expressed and reported; thus, simple translation is insufficient cultural adaptation and psychometric validation are required to ensure reliability and validity in the target population.

The present study aimed to translate and culturally adapt the EBA-O into Persian and evaluate its psychometric properties, including factorial structure, reliability, and validity in an Iranian population. We hypothesized that the Persian version would replicate the original five-factor structure and demonstrate internal consistency and fit indices comparable to those reported in the original and Greek validations. Establishing a validated Persian EBA-O will address an important gap by enabling the assessment of pathological eating behaviors in Persian-speaking individuals with obesity, thereby supporting more precise phenotypic characterization and improved obesity-management strategies.

## 2. Methods

### 2.1. Translation and Cultural Adaptation

Permission was obtained from the original developers of the EBA-O to translate the instrument. We followed a standardized forward–backward translation protocol to ensure cross-cultural appropriateness. First, two independent bilingual translators—both fluent in English and Persian, with one having a background in medical psychology—produced separate forward translations of the questionnaire. The translators and the research team then compared, discussed, and reconciled the two versions into a single provisional Persian translation, resolving discrepancies through consensus. Particular attention was given to preserving the conceptual meaning of terms describing pathological eating behaviors (e.g., night eating, hyperphagia, food cravings) in a manner easily understandable to Persian-speaking laypersons.

Next, a third bilingual translator, blinded to the original questionnaire, back-translated the reconciled Persian version into English. The research team, including an expert in eating disorders, carefully reviewed the back-translation alongside the original EBA-O to ensure conceptual equivalence, clarity, and linguistic accuracy. Minor wording adjustments were made to enhance cultural relevance, such as refining the item referring to the consumption of sweet foods or sugary drinks when sad or anxious, using examples familiar within the Iranian context.

An expert panel consisting of psychologists and nutritionists reviewed the pre-final Persian version to evaluate face validity and cultural suitability. The questionnaire was then pilot-tested in a small sample of 30 adults with obesity to assess comprehension, clarity, and interpretability. Feedback indicated that all items were well understood, and therefore no further modifications were required, leading to the finalization of the Persian EBA-O.

The response options and scoring system remain identical to the original version: participants indicate the frequency of each behavior during the past three months on an 8-point Likert-type scale ranging from 0 (“never”) to 7 (“every day”). Subscale scores are calculated as the mean of the corresponding items, and the total score is computed as the average of all items—or equivalently, the mean of the five subscale scores [12]. In accordance with the original authors’ guidelines, a subscale or total score of 4 or higher indicates a clinically meaningful level of that eating behavior [12].

### 2.2. Participants and Procedure

Participants were recruited from the general population as well as outpatient weight-management clinics in Tehran. A convenience sampling approach was used to enroll individuals between July 2023 and February 2024. The study was advertised through clinic notice boards and social media posts targeting adults interested in weight loss or nutrition counseling.

Inclusion criteria were: (a) age ≥ 18 years; (b) ability to read and write Persian; (c) BMI ≥ 25 kg/m^2^; and (d) ability to understand and complete self-report questionnaires and provide informed consent. Individuals with cognitive impairments or severe psychiatric conditions that could interfere with independent questionnaire completion were excluded.

All participants provided written informed consent after receiving a full explanation of the study objectives and confidentiality procedures. The study protocol was approved by the Ethics Committee of Mashhad University of Medical Sciences (IR.MUMS.MEDICAL.REC.1402.480).

To ensure adequate statistical power for scale validation, we aimed to recruit a minimum of 300 participants, consistent with methodological recommendations [13,14,15]. After providing consent, participants completed a brief demographic form (assessing age, sex, education, and self-reported height and weight for BMI calculation), the Persian EBA-O, and additional psychometric measures used to evaluate convergent validity, including the Three-Factor Eating Questionnaire–R18 (TFEQ-R18), the Binge Eating Severity Questionnaire, and the Food Addiction Questionnaire, all validated for Persian-speaking populations [16,17,18].

A subset of participants (*n* = 40) completed the Persian EBA-O again after a two-week interval to assess test–retest reliability [19]. Participants completed the questionnaires either on paper at clinic sites or online through a secure web-based survey. For those completing the forms in clinical settings, responses were reviewed by researchers to ensure completeness. Online submissions underwent post hoc eligibility screening, and cases that did not meet inclusion criteria or demonstrated invalid responding were removed.

### 2.3. Statistical Analysis

Descriptive analyses were conducted to summarize participants’ demographic and clinical characteristics (including age, sex, BMI, marital status, education level, and general health status), as well as the total and subscale scores of the Persian EBA-O. Because the data did not follow a normal distribution, continuous variables were presented as median and interquartile range (IQR 25–75), while categorical variables were reported as frequencies and percentages.

We performed a confirmatory factor analysis (CFA) to examine whether the Persian EBA-O fit the expected five-factor structure of the original instrument. Although the EBA-O can be modeled as a second-order structure, we tested a first-order model to evaluate the distinct behavioral dimensions individually. Data normality was assessed prior to conducting the analyses. The five latent factors (Food Addiction, Night Eating, Binge Eating, Sweet Eating, and Hyperphagia) were specified according to the original factor groupings.

CFA was conducted using Partial Least Squares Structural Equation Modeling (PLS-SEM) in SmartPLS version 4, based on data from 287 participants. Model fit was evaluated through multiple fit indices: the Comparative Fit Index (CFI) and Tucker–Lewis Index (TLI), where values ≥ 0.90 indicate acceptable fit (≥0.95 excellent fit); the Normed Fit Index (NFI) and Goodness-of-Fit Index (GFI), with values ≥ 0.90 considered good; the Standardized Root Mean Square Residual (SRMR), where values < 0.08 denote good fit; and the relative chi-square (χ^2^/*df*), with values < 3 indicating acceptable fit and <2 indicating very good fit. These indices are presented to facilitate comparison with previous validation studies. Because the chi-square statistic is highly sensitive to sample size, greater emphasis was placed on relative chi-square and approximate fit indices (CFI, TLI, etc.).

To assess internal consistency reliability, we calculated Cronbach’s alpha for each of the five subscales as well as the total scale. Although McDonald’s omega is often considered a more precise reliability estimator, Cronbach’s alpha and Composite Reliability (CR) were deemed sufficient for the purposes of this study. CR was computed for each factor as a SEM-based reliability index analogous to coefficient omega. Values of α and CR ≥ 0.70 were interpreted as satisfactory reliability. Bootstrap 95% confidence intervals (5000 resamples) were calculated to assess the precision of these estimates.

Convergent validity was examined using the Average Variance Extracted (AVE), which represents the average proportion of variance explained by each factor. An AVE ≥ 0.50 was considered evidence of adequate convergent validity. Factors with strong reliability (α, CR) but AVE slightly below 0.50 were still considered acceptable when supported by other validity evidence.

Discriminant validity among the five factors was evaluated using the Heterotrait–Monotrait (HTMT) ratio of correlations. HTMT reflects the ratio of average correlations between constructs to the average correlations within constructs. HTMT values < 0.90 were considered indicative of distinct constructs, whereas values > 0.90 suggest inadequate discriminant validity. HTMT values were computed for all factor pairs along with bootstrap 95% CIs.

To further examine convergent validity, Pearson correlation coefficients were used to evaluate associations between total EBA-O scores and established measures of disordered eating. Independent samples t-tests were used for group comparisons, and intraclass correlation coefficients (ICCs) along with paired *t*-tests were used to evaluate two-week test–retest reliability.

All statistical analyses were conducted using SPSS version 26 and SmartPLS version 4. Because the primary objective of the study was to validate an existing factor structure, exploratory factor analysis was not performed. Missing data were not imputed because item non-response was minimal (<1%). Cases with missing values were handled using pairwise deletion when necessary. The figure was created using PLS4 (Partial Least Squares, version 4).

## 3. Results

### 3.1. Participant Characteristics

After data cleaning—including removal of cases with excessive missing values or implausible responses—a total of 287 participants were retained for analysis. All respondents met the age and BMI inclusion criteria, so no exclusions were necessary based on these variables. The demographic and clinical characteristics of the sample are summarized in Table 1.

The median age of participants was 36.0 years (IQR: 31.0–40.0). The sample was predominantly female, consisting of 239 women (83.3%) and 48 men (16.7%). Regarding marital status, 189 individuals (65.9%) were married, while 98 (34.1%) were single.

The median BMI was 32.84 kg/m^2^ (IQR: 31.22–35.98), indicating that the majority of participants were classified as obese. The median body weight was 90.0 kg (IQR: 83.0–103.0), and the median height was 165.0 cm (IQR: 160.0–170.0).

In terms of educational attainment, most respondents held a bachelor’s degree (*n* = 164, 57.1%), followed by those with a master’s degree or higher (*n* = 63, 22.0%). The remainder included participants with a diploma (*n* = 52, 18.1%) and those currently completing undergraduate studies (*n* = 8, 2.8%).

Regarding self-reported health status, 41.8% of the sample reported having no chronic medical conditions. Among those reporting a health issue, 32.8% indicated symptoms of depression or anxiety, 16.4% reported fatty liver disease, 3.8% had diabetes, 1.7% had cardiovascular disease, 2.4% reported kidney disease, and 27.2% indicated other health conditions.

### 3.2. Factor Structure (Confirmatory Factor Analysis)

CFA was conducted to evaluate the extent to which the Persian version of the EBA-O replicated the expected five-factor latent structure. The model specified five correlated factors: Food Addiction, Night Eating, Binge Eating, Sweet Eating, and Hyperphagia, each represented by their respective item subsets.

All 18 items loaded significantly onto their intended factors (*p* < 0.001). Standardized factor loadings ranged from moderate to high, closely reflecting the pattern reported in the original validation study. Items belonging to the Sweet Eating factor demonstrated strong loadings (approximately 0.70–0.80), particularly those assessing cravings for sweets in response to emotional distress. Likewise, the Binge Eating items showed robust loadings (around 0.80). The Food Addiction factor exhibited greater variability in item loadings, with several loadings exceeding 0.80 (e.g., difficulty stopping consumption of specific foods), whereas one item related to irritability or withdrawal-like symptoms when reducing intake showed a more modest loading (~0.55–0.60), a pattern consistent with the original EBA-O findings. Items assessing Night Eating showed loadings ranging from approximately 0.65 to 0.80.

Overall, model fit indices indicated that the five-factor structure demonstrated an acceptable to good fit. The model achieved a CFI of 0.95 and a TLI of 0.94, both exceeding the recommended minimum threshold of 0.90. Additional indices further supported model adequacy, including NFI = 0.91 and GFI = 0.92. The SRMR value of 0.05 was well below the maximum acceptable cutoff of 0.08. The relative chi-square (χ^2^/*df* = 1.94) also reflected a good fit, remaining below the conventional benchmark of 3. Taken together, these metrics confirm that the Persian EBA-O aligns well with the originally hypothesized model.

**Figure 1:**
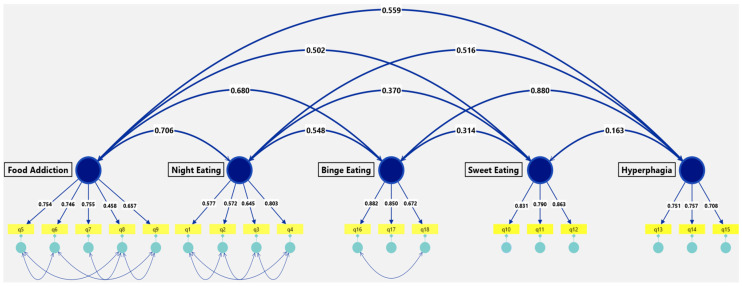
Confirmatory factor analysis (CFA) model. The model illustrates significant correlations among five overeating-related constructs, each measured by a set of observed variables.

As expected, the five latent factors were significantly intercorrelated (Figure 1), reflecting their conceptual overlap within the domain of pathological overeating. The strongest correlation was observed between Binge Eating and Hyperphagia (r = 0.880). Food Addiction showed moderately high associations with both Binge Eating (r = 0.680) and Hyperphagia (r = 0.559). Night Eating demonstrated moderate correlations with Food Addiction (r = 0.706) and Binge Eating (r = 0.548) but weaker relationships with Sweet Eating (r = 0.370) and Hyperphagia (r = 0.516). The Sweet Eating factor showed a modest correlation with Binge Eating (r = 0.314), a weak correlation with Night Eating (r = 0.370), and minimal overlap with Hyperphagia (r = 0.163).

### 3.3. Reliability and Convergent Validity

Composite reliability (CR) coefficients closely paralleled the Cronbach’s α results: all five factors demonstrated CR values above the recommended threshold of 0.70, ranging approximately from 0.71 to 0.86 (Table 2). For instance, the Sweet Eating factor showed CR = 0.86, Binge Eating CR = 0.84, Hyperphagia CR = 0.78, Food Addiction CR = 0.76, and Night Eating CR = 0.71. These CR values confirm that each item set reliably captures its respective latent construct. Notably, Night Eating had the lowest CR (≈0.71), consistent with its Cronbach’s α of 0.78, indicating adequate but comparatively weaker internal cohesion relative to subscales such as Sweet Eating (α = 0.86, CR = 0.86).

Convergent validity was assessed using the Average Variance Extracted (AVE) for each factor. Three of the five factors surpassed the conventional AVE threshold of 0.50, indicating that these constructs explain at least half of the variance in their corresponding items. Specifically, Sweet Eating demonstrated a high AVE of 0.68, and Binge Eating an AVE of 0.65, suggesting that their items are highly cohesive and strongly representative of their intended domains. Hyperphagia also achieved an acceptable AVE of 0.54, slightly above the benchmark.

In contrast, the Food Addiction factor showed an AVE of 0.46, and Night Eating an AVE of 0.43, both slightly below the 0.50 guideline. This suggests that, on average, less than half of the variance in their items is accounted for by the latent factor. Despite this, both subscales exhibited sound reliability, as evidenced by Cronbach’s α and CR values well above 0.70. This pattern indicates that the subscales still measure coherent constructs, although one or two items with lower factor loadings may be contributing to the lower AVE values.

For example, the Food Addiction subscale includes an item assessing withdrawal-like symptoms (e.g., irritability or headaches when reducing intake), which showed a lower loading (~0.56) in our dataset—consistent with findings from the original scale. Similarly, in the Night Eating subscale, the item regarding eating to fall back asleep is not uniformly endorsed across night eaters, which may reduce the average variance extracted.

Because the AVE values for Food Addiction and Night Eating were only marginally below the recommended 0.50 threshold and given their adequate CR values and strong internal consistency, we did not consider these findings to represent a meaningful threat to convergent validity.

### 3.4. Test–Retest Reliability

The test–retest reliability over a two-week interval (*n* = 40) was acceptable, with ICCs for all subscales exceeding 0.70. Pearson correlation coefficients between baseline and retest scores were also computed, showing strong associations and indicating no significant score differences across the two administrations.

### 3.5. Discriminant Validity

Discriminant validity among the five EBA-O factors was supported by multiple criteria. The more stringent HTMT ratio analysis confirmed that discriminant validity was satisfactory. Table 3 presents HTMT values for each factor pair along with their 95 percent confidence intervals. All HTMT values were below the conservative 0.90 threshold. The highest HTMT value was observed between the Binge Eating and Hyperphagia factors (HTMT = 0.89, 95% CI [0.82, 0.96]). Although this value is near the cutoff, it remains below 0.90, indicating that binge eating and general overeating tendencies, while conceptually related, are still statistically distinguishable constructs. The Food Addiction factor showed an HTMT of approximately 0.69 with Binge Eating and around 0.53 with Hyperphagia, suggesting that addictive-like eating urges, despite their correlation with overeating, represent a distinct dimension. The Night Eating factor demonstrated HTMT values of roughly 0.65 with Food Addiction and 0.52 with Sweet Eating, all comfortably below 0.90.

### 3.6. Convergent Validity Analyses

#### 3.6.1. Three-Factor Eating Questionnaire-R18 (TFEQ-R18)

The total EBA-O score showed a moderate, positive correlation with the total TFEQ-R18 score (r = 0.517, *p* < 0.001), indicating that individuals who report higher levels of maladaptive eating behaviors on the EBA-O also tend to exhibit greater disinhibition and emotional eating tendencies. This pattern supports the convergent validity of the Persian EBA-O.

#### 3.6.2. Binge Eating Severity Questionnaire

A strong positive correlation was found between the EBA-O total score and the Binge Eating Severity Questionnaire (r = 0.713, *p* < 0.001), providing additional evidence of convergent validity. This strong association indicates that individuals with elevated scores on the EBA-O also report more severe binge-eating symptoms, consistent with the theoretical overlap between the constructs measured by these instruments.

#### 3.6.3. Food Addiction Questionnaire

Independent samples t-tests further supported convergent validity. Individuals classified as having food addiction scored significantly higher on the Food Addiction subscale of the EBA-O (M = 3.48, SD = 1.55) compared with those not classified as food-addicted (M = 1.93, SD = 1.25), t (285) = −9.28, *p* < 0.001.

## 4. Discussion

This study set out to translate, culturally adapt, and validate the EBA-O questionnaire in the Persian language. The findings provide strong evidence that the Persian EBA-O is psychometrically sound and retains the theoretical five-factor structure of its original instrument. To our knowledge, this is the first validation of the EBA-O in a non-European language, following its initial development in English and Italian and the more recent validation in Greek. Our results align with prior studies and offer several important insights.

**Factor Structure and Model Fit:** The confirmatory factor analysis confirmed the presence of five distinct yet related domains: Food Addiction, Night Eating, Binge Eating, Sweet Eating, and Hyperphagia. The fit indices (CFI = 0.95, TLI = 0.94, etc.) indicated that the hypothesized model provides an excellent representation of the data. This level of fit is comparable to—and in some aspects slightly better than—the original validation (CFI = 0.93) [12], and is close to the near-perfect fit reported in the Greek sample (CFI = 0.99) [11]. Minor variations in model fit may reflect differences in sample composition or estimation methods. Nevertheless, the Persian CFA demonstrates that the EBA-O’s factor structure is applicable across cultures.

We intentionally constrained our analysis to the same structure defined by Segura-García et al. [12], and no model modifications (e.g., correlated error terms or cross-loadings) were required, underscoring the robustness of the instrument’s design. However, some factors—particularly Binge Eating and Hyperphagia—were highly correlated (r = 0.880). This strong association may indicate a shared underlying component, such as impulsivity or loss of control, common to both behaviors. From a theoretical perspective, this supports the view that overeating behaviors in obesity may exist on a continuum rather than as strictly discrete phenomena [20]. This pattern is also consistent with clinical expectations and mirrors findings from the Greek validation (“the factors were highly correlated, with the strongest correlation between factors 3 and 5,” corresponding to binge eating and hyperphagia). High correlations among EBA-O subscales likely reflect the overlapping nature of overeating behaviors; individuals who consume unusually large portions at meals often also report episodes of loss of control (binging), etc. [21]. Importantly, despite these strong associations, our discriminant validity tests (HTMT ratios) remained below the recommended threshold of 0.90, indicating that the subscales are related but non-redundant constructs. This balance suggests that the instrument captures a coherent overarching construct (pathological eating in obesity) while preserving meaningful sub-dimensions.

The preservation of the five-factor structure across cultures suggests that maladaptive eating patterns—such as binge eating, sweet eating, and night eating—may reflect universal behavioral phenotypes within obesity. This cross-cultural consistency supports theoretical frameworks that conceptualize overeating as a multidimensional construct influenced by biological, emotional, and environmental factors rather than as culturally bound syndromes [22,23].

**Reliability:** The Persian EBA-O demonstrated high internal consistency, with Cronbach’s α values ranging from 0.78 to 0.86 across subscales. These values closely align with those reported in other versions of the questionnaire. For example, the original English EBA-O reported McDonald’s ω between 0.80 and 0.92 across factors [12], which is comparable to our α estimates, while the Greek version reported ω values of 0.82–0.92 for its subscales [11]. The slightly lower reliability observed for the Night Eating subscale (α = 0.78) may reflect cultural or sample-specific differences in the expression of night eating behaviors, or it may be due to the small number of items in this subscale (four items), which can inherently limit alpha. Nonetheless, α = 0.78 is considered acceptable for research purposes and is similar to the Greek validation (ω = 0.82). Composite reliability metrics further confirmed that each Persian EBA-O subscale consistently measured its intended construct. Collectively, these reliability indicators suggest that the translation did not introduce ambiguity or inconsistency, and that Persian-speaking respondents interpreted the items in a coherent manner comparable with respondents in other cultural contexts.

Test–retest reliability analyses further supported the instrument’s temporal stability. All subscales demonstrated ICCs greater than 0.70 over a two-week interval, indicating satisfactory reproducibility. The lack of significant differences between baseline and follow-up scores suggests that the underlying constructs remain stable over short periods and are not overly influenced by temporary emotional or situational factors. These results support the use of the EBA-O in both clinical and research settings where repeated assessment may be required.

The consistently high internal consistency across subscales indicates that participants understood and interpreted the items cohesively. Practically, this means that the Persian EBA-O can reliably differentiate between distinct overeating behaviors, an essential feature for tailoring individualized treatment strategies that target specific domains of eating pathology.

**Convergent and Discriminant Validity:** Convergent validity was strongly supported for three of the five factors, each demonstrating AVE values well above 0.50. Convergent validity was somewhat weaker for the Food Addiction and Night Eating factors, which showed AVE values in the mid-0.40s. This pattern may reflect inherent conceptual heterogeneity within these constructs. Food Addiction, for instance, is a broad domain encompassing multiple symptoms analogous to substance addiction—such as craving, continued use despite negative consequences, unsuccessful attempts to cut down, and withdrawal-like symptoms [24,25]. Not all individuals who score high on food addiction endorse these features to the same degree; for example, some may report intense cravings and loss of control without experiencing withdrawal symptoms, or vice versa.

A similar explanation applies to the Night Eating factor. Night Eating Syndrome represents a cluster of behaviors—including nocturnal ingestion, difficulty returning to sleep without eating, and pronounced evening hyperphagia—and individuals may vary in which of these features they exhibit [26,27]. For instance, one person may frequently eat late at night but never wake from sleep to eat, whereas another may show the opposite pattern. Consequently, the items within the Night Eating domain, although conceptually coherent, may not intercorrelate as strongly as those within more uniform constructs such as Sweet Eating, where all items revolve around a single behavioral theme.

Despite AVE values slightly below 0.50 for these two subscales, we retained all original items to preserve the full conceptual scope of each construct. Composite reliability and item loadings indicated that no item was problematic or detracted from the integrity of the subscale. Future studies may refine these domains—such as by adding items that capture additional aspects of food addiction or night eating—to further enhance variance explained. Nonetheless, the present results indicate that the Persian EBA-O adequately represents the intended constructs.

Discriminant validity was well supported, as no pair of factors demonstrated excessive overlap. HTMT analyses were particularly reassuring; all HTMT values were below the recommended cutoff of 0.90, with most well below this threshold. This indicates that participants were able to distinguish between, for example, eating at night versus daytime binge episodes, or addictive cravings versus general overeating at meals.

These findings parallel results from the Greek validation, where factors were also noted to be highly interrelated yet sufficiently distinct to warrant separate measurement. In our dataset, the borderline-high correlation between Binge Eating and Hyperphagia likely reflects true behavioral co-occurrence—individuals who frequently consume excessively large meals often also experience episodes of loss-of-control eating. Still, empirical indicators confirmed that these remain separable constructs for assessment purposes.

The borderline case was between the Binge Eating and Hyperphagia factors (HTMT = 0.89), reflecting their close conceptual relationship. The Greek validation study similarly noted that these behaviors—as well as sweet-eating—tended to cluster together within their sample. Despite this overlap, the ability of the EBA-O to meaningfully distinguish between these constructs is clinically advantageous. For example, interventions for an individual whose primary difficulty is structured hyperphagia (i.e., consistently large portion sizes during meals) may differ from those required for someone experiencing intermittent binge-eating episodes, even though these patterns frequently co-occur. Our findings indicate that the EBA-O is capable of capturing such nuances.

Retaining Hyperphagia as an independent factor is therefore a strength of the instrument, and our data support its distinctiveness as intended. In summary, the pattern of inter-factor correlations in the Persian sample closely mirrors both prior findings and theoretical expectations: these maladaptive eating behaviors represent interrelated yet non-redundant facets of the broader construct of overeating in obesity.

Furthermore, our convergent validity analyses using established external measures provide additional support for the construct validity of the Persian EBA-O. The moderate correlation with the TFEQ-R18 (r = 0.517, *p* < 0.001) underscores the relationship between general maladaptive eating tendencies and the specific behavioral domains assessed by the EBA-O. The strong association with the Binge Eating Severity Questionnaire (r = 0.713, *p* < 0.001) further confirms the instrument’s sensitivity to binge-eating pathology, one of the central eating-behavior domains in obesity. Additionally, the significant difference in Food Addiction subscale scores between individuals with and without food addiction diagnoses (mean difference = 1.54, *p* < 0.001) provides compelling evidence of discriminative validity. Collectively, these findings align with theoretical expectations and support the EBA-O’s utility in identifying behaviorally distinct subgroups among individuals with obesity.

**Comparison with Greek and Original Validations:** Overall, our findings are consistent with both the Greek validation by Mavrandrea et al. [11] and the original development study by Segura-García et al. [12]. All three versions (original, Greek, and Persian) support the same five-factor structure and demonstrate strong reliability. A minor point of divergence is that the Greek CFA reported exceptionally high fit indices (CFI/TLI = 0.99). This may be attributable to sample characteristics—the Greek sample was slightly smaller after excluding individuals with BMI < 25 (N = 223), and smaller samples can occasionally inflate fit indices when models include numerous parameters. Differences in estimation techniques may also have contributed, although the Greek article does not specify the exact method. In contrast, our CFI of 0.95 represents an excellent fit and is arguably more realistic for a multifactorial behavioral measure. The SRMR of 0.05 in our model further indicates minimal unexplained covariance, which is a positive indicator of model adequacy. In the original study, the CFA reported an RMSEA of approximately 0.075, suggesting moderate misfit, possibly due to methodological or sample differences. By comparison, our SRMR and relative χ^2^ (1.94) suggest a tighter-fitting model for the Persian version. Thus, the Persian EBA-O appears to perform at least as well as the original in terms of structural integrity and nearly as well as the Greek version in overall model fit.

In terms of reliability, the Greek study reported a total scale ω of 0.94, which aligns closely with our total α of 0.89, especially considering that ω often slightly exceeds α for multidimensional scales. Subscale reliability values across the original, Greek, and Persian versions were also comparable, indicating that the consistency of each domain is preserved across translations. The Greek study further supported concurrent validity by correlating EBA-O scores with external measures such as the EDE-Q, BES, and YFAS, demonstrating meaningful associations with established instruments.

Although we included several relevant measures for convergent validity (e.g., TFEQ-R18, Food Addiction Questionnaire, and Binge Eating Severity Questionnaire), the number of external validation instruments was limited due to the desire to reduce respondent burden and the limited availability of validated Persian tools at the time. This is a limitation of the present study, as we could not provide a more extensive evaluation of external convergent validity. Nonetheless, because the Persian EBA-O’s structural and psychometric characteristics closely mirror those of the original and Greek versions, it is reasonable to expect that future studies—including instruments such as the Persian Binge Eating Scale or the Persian Three-Factor Eating Questionnaire—will demonstrate similar validity patterns.

Taken together, our findings indicate that the Persian EBA-O preserves the structural and psychometric integrity of the original instrument. The observed overlap between subscales such as binge eating and hyperphagia may reflect the inherent complexity of obesity-related eating behaviors, where boundaries between pathological and normative overeating can be fluid. Future research should further explore whether these behavioral domains predict distinct metabolic or psychological outcomes.

**Cultural Considerations:** One of the primary aims of this study was to ensure that the EBA-O was not merely translated but also culturally adapted for Persian-speaking populations. During the translation process, we found that most questionnaire items were inherently applicable to Persian culture and required only minimal adjustments. Overeating behaviors—such as late-night snacking, cravings for high-fat and high-carbohydrate foods, and consuming large portions—are common across cultures, including Iran. Minor wording modifications were made to improve clarity, such as adding culturally relevant examples of high-carbohydrate foods (e.g., rice or bread, which are dietary staples in Iran) to items addressing food cravings.

Although night eating syndrome is not widely recognized as a clinical entity in Iran, late-night eating is culturally common, given the tendency toward later dinner times. The distribution of item responses in our sample confirmed that nocturnal eating behaviors were endorsed by a subset of participants. Thus, the content validity of the Persian version appears to remain intact. While we did not formally assess linguistic equivalence beyond the back-translation procedure, the strong psychometric performance suggests that any linguistic nuances did not hinder comprehension.

**Clinical and Research Implications:** The successful validation of the Persian EBA-O provides clinicians and researchers working with Persian-speaking individuals a comprehensive and practical tool for assessing problematic eating behaviors associated with obesity. This scale can be particularly valuable in bariatric surgery evaluations, structured weight-management programs, and obesity clinics across Iran and neighboring regions. With the EBA-O, clinicians can efficiently screen for behaviors such as night eating or binge eating, which may otherwise go undetected. Recognizing these behaviors is essential, as individuals with untreated binge eating or night eating tendencies often demonstrate poorer responses to standard weight-loss interventions and may require targeted psychotherapeutic or pharmacological support.

The EBA-O also aids in delivering tailored interventions. For example, individuals scoring high on Sweet Eating may benefit from strategies to manage emotional cravings for sweets, whereas those with elevated Food Addiction or Binge Eating scores may require cognitive–behavioral approaches focused on impulse control and coping skills. Importantly, because the EBA-O is easy to administer and does not require specialized training, it can be used by nutritionists, general practitioners, and other healthcare professionals. This is particularly advantageous in Iran, where access to eating-disorder specialists may be limited.

From a research standpoint, the Persian EBA-O enables rigorous investigation of obesity phenotypes in Iran. Researchers can examine the prevalence of maladaptive eating patterns such as night eating syndrome or food addiction and explore their relationships with metabolic outcomes, psychological factors, and treatment responses. The validated scale also facilitates epidemiological studies aimed at understanding the broader distribution of these behaviors within the general population and identifying cultural or environmental risk factors. Additionally, the availability of this tool opens opportunities for cross-cultural comparisons to determine whether patterns observed among Iranian individuals with obesity differ from those reported in other countries. Preliminary impressions suggest substantial overlap, pointing to the possibility that many overeating behaviors may be universal rather than culture-specific, though subtle distinctions (e.g., portion norms) warrant exploration.

## 5. Strengths and Limitations

This study possesses several strengths, including a rigorous and well-documented translation and back-translation protocol, an adequate sample size (*n* = 287) for confirmatory factor analysis and reliability testing, and the application of comprehensive modern statistical methods—such as HTMT—to evaluate psychometric properties. The alignment of our findings with previous validations further supports the robustness of the Persian EBA-O. The study additionally demonstrated strong convergent validity through meaningful correlations with established measures of binge eating, emotional eating, and food addiction, as well as satisfactory test–retest reliability over a two-week interval.

Nonetheless, certain limitations should be acknowledged. The sample was not fully representative of the broader Iranian population affected by overweight and obesity; participants were predominantly female, relatively young, and highly educated. This may limit generalizability to other groups such as men, older adults, and individuals with lower educational attainment. Future studies should aim to recruit more diverse samples to confirm the scale’s applicability across sociodemographic strata. Additionally, because only a limited number of validated Persian instruments were available, external convergent and criterion validity could not be comprehensively assessed. Cross-cultural measurement invariance was also not tested and should be addressed in future research using multi-group CFA. Finally, while the EBA-O effectively captures five clinically meaningful eating-behavior domains, it does not cover all aspects of eating psychopathology (e.g., emotional eating, grazing) and may benefit from complementary tools when broader assessment is needed.

## 6. Conclusions

The Persian version of the EBA-O demonstrated robust psychometric properties, successfully replicating the original five-factor structure and confirming its reliability and validity among Iranian adults with overweight and obesity. Consistent with prior validations, our findings underscore the instrument’s cross-cultural applicability in assessing key maladaptive eating behaviors linked to obesity. By filling an important methodological gap, the validated Persian EBA-O equips clinicians and researchers with a culturally relevant and evidence-based tool for identifying eating-behavior phenotypes such as night eating and binge eating in Persian-speaking populations. This, in turn, supports more precise, individualized, and effective intervention strategies. Future research should explore predictive validity and responsiveness to intervention; nonetheless, the current evidence positions the Persian EBA-O as a valuable asset for improving obesity assessment and care in Iran.

## Figures and Tables

**Table 1:** Demographic data of samples.

Variables	Median [IQR 25–75)/Frequency (n, %)
Age (year) ^a^	36.0 [31.0–40.0]
**Sex**	
Female (n, %)	239 (83.3%)
Male (n, %)	48 (16.7%)
**Marital status**	
Single (n, %)	98 (34.1%)
Married	189 (65.9%)
Body mass index (kg/m^2^) ^a^	32.84 [31.22–35.98]
Weight (kg) ^a^	90.0 [83.0–103.0]
Height (cm) ^a^	165.0 [160.0–170.0]
**Education**	
Undergraduate (n, %)	8 (2.8%)
Diploma (n, %)	52 (18.1%)
Bachelor’s degree (n, %)	164 (57.1)
Master’s degree and upper (n, %)	63 (22.0%)
**Diseases**	
No diseases (n, %)	120 (41.8)
Depression and anxiety (n, %)	94 (32.8%)
Fatty Liver (n, %)	47 (16.4%)
Diabetes (n, %)	11 (3.8%)
Cardiovascular diseases (n, %)	5 (1.7%)
Kidney diseases (n, %)	7 (2.4%)
Others (n, %)	78 (27.2%)

^a^ As data had a non-normal distribution, they were presented as Median [IQR 25–75].

**Table 2:** Construct Reliability and Convergent Validity Assessment.

	Food Addiction (CI)	Night Eating (CI)	Binge Eating (CI)	Sweet Eating (CI)	Hyperphagia (CI)	Acceptable Range
**Cronbach’s α**	0.82 (0.78, 85)	0.77 (0.71, 0.82)	0.82 (0.77, 0.86)	0.86 (0.83, 0.89)	0.78 (0.72, 0.83)	>0.7
**CR**	0.76 (0.70, 81)	0.71 (0.61, 0.83)	0.84 (0.79, 0.89)	0.86 (0.83, 0.89)	0.78 (0.72, 0.83)	>0.7
**AVE**	0.46 (0.40, 053)	0.43 (0.34, 0.53)	0.65 (0.58, 0.71)	0.68 (0.62, 0.74)	0.54 (0.46, 0.62)	>0.5

Abbreviations: CR: Composite reliability; AVE: Average Variance Extracted.

**Table 3:** Discriminant validity-Heterotrait–monotrait ratio (HTMT).

	Food Addiction (CI)	Night Eating (CI)	Binge Eating (CI)	Sweet Eating (CI)	Hyperphagia (CI)
**Food addiction**	-	-	-	-	-
**Night eating**	0.65 (0.53, 0.75)	-	-	-	-
**Binge eating**	0.69 (0.57, 0.79)	0.53 (0.40, 0.65)	-	-	-
**Sweet eating**	0.52 (0.40, 0.64)	0.33 (0.20, 0.46)	0.37 (0.24, 0.50)	-	-
**Hyperphagia**	0.53 (0.40, 0.65)	0.50 (0.36, 0.62)	0.89 (0.82, 0.96)	0.18 (0.10, 0.31)	-

## Data Availability

The datasets generated and/or analyzed during the current study are available from the corresponding author pon reasonable request due to ethical and privacy considerations.
